# Artificial Intelligence in Multilingual Interpretation and Radiology Assessment for Clinical Language Evaluation (AI-MIRACLE)

**DOI:** 10.3390/jpm14090923

**Published:** 2024-08-30

**Authors:** Praneet Khanna, Gagandeep Dhillon, Venkata Buddhavarapu, Ram Verma, Rahul Kashyap, Harpreet Grewal

**Affiliations:** 1 The University of Missouri-Kansas City School of Medicine, Kansas City, MO 64108, USA; 2 Department of Internal Medicine, University of Maryland Baltimore Washington Medical Center, Glen Burnie, MD 21061, USA; gagandeep.dhillon@umm.edu; 3 Banner Baywood Medical Center, Banner Health, Mesa, AZ 85206, USA; venkatasrihari.buddhavarapu@bannerhealth.com; 4 Department of Sleep Medicine, Parkview Health System, Fort Wayne, IN 46845, USA; 5 Department of Research, WellSpan Health, York, PA 17403, USA; kashyapmd@gmail.com; 6 Division of Pulmonary and Critical Care Medicine, Mayo Clinic, Rochester, MN 55905, USA; 7 Department of Radiology, College of Medicine, Florida State University, Pensacola, FL 32514, USA

**Keywords:** artificial intelligence, radiology, translation, ChatGPT 4.0

## Abstract

The AI-MIRACLE Study investigates the efficacy of using ChatGPT 4.0, a large language model (LLM), for translating and simplifying radiology reports into multiple languages, aimed at enhancing patient comprehension. The study assesses the model’s performance across the most spoken languages in the U.S., emphasizing the accuracy and clarity of translated and simplified radiology reports for non-medical readers. This study employed ChatGPT 4.0 to translate and simplify selected radiology reports into Vietnamese, Tagalog, Spanish, Mandarin, and Arabic. Hindi was used as a preliminary test language for validation of the questionnaire. Performance was assessed via Google form surveys distributed to bilingual physicians, which assessed the translation accuracy and clarity of simplified texts provided by ChatGPT 4. Responses from 24 participants showed mixed results. The study underscores the model’s varying success across different languages, emphasizing both potential applications and limitations. ChatGPT 4.0 shows promise in breaking down language barriers in healthcare settings, enhancing patient comprehension of complex medical information. However, the performance is inconsistent across languages, indicating a need for further refinement and more inclusive training of AI models to handle diverse medical contexts and languages. The study highlights the role of LLMs in improving healthcare communication and patient comprehension, while indicating the need for continued advancements in AI technology, particularly in the translation of low-resource languages.

## 1. Introduction

Artificial intelligence (AI) is increasingly becoming more integrated into healthcare settings, offering innovative solutions across various domains, including diagnostics and patient communications. Chat Generative Pre-training Transformer (ChatGPT™), a large language learning model (LLM) developed by OpenAI in San Francisco, California, USA [1], is designed to processes extensive datasets of text and code. It has the capability to generate text, translate languages, write different kinds of creative content, and provide informative answers to questions [2]. There are several other LLMs such as Gemini [3], Claude [4], and Bard [5] available to use, but ChatGPT, one of the older and more widely used LLMs, is a natural choice for studying the translation of radiology reports into different languages, particularly due to the broader availability of its free version. This study is among the first to explore the potential of ChatGPT 4.0 in breaking language barriers in healthcare communication, especially in an era when patients have direct access to their radiology reports via patient portals. The study assesses the utility of ChatGPT in enhancing patient understanding and engagement through accurate translations of medical reports.

### Background

Radiology is at the forefront of adoption of AI and LLMs, although there is widespread usage across various medical subspecialities, such as critical and emergency medicine [6] and sleep medicine [7]. Radiology reports are an integral part of clinical decision-making, and their technical jargon and complex language can pose significant barriers to patient comprehension, especially for patients without medical training [8]. This communication gap is particularly pronounced in multilingual settings, where language differences further complicate the effective dissemination of critical health information [9]. The “Artificial Intelligence in Multilingual Interpretation and Radiology Assessment for Clinical Language Evaluation”, (AI-MIRACLE study), presents a novel approach in leveraging advanced large language models (LLMs), such as ChatGPT 4.0, to address an important challenge in healthcare communication: the translation and simplification of radiology reports. The primary objective of the AI-MIRACLE study is to evaluate the performance of ChatGPT 4.0 in both translating and simplifying radiology reports, thereby enhancing patient comprehension. By assessing the accuracy and clarity of these translations and simplified texts, the study seeks to determine the potential applications and limitations of current AI technologies in addressing the multilingual needs of healthcare settings. The findings of this study could inform future developments in AI technology, particularly in the improvement of LLMs for medical applications, and lay the groundwork for more innovative healthcare communication practices.

## 2. Materials and Methods

The AI-MIRACLE study is a cross-sectional study for validating the application of large language models (LLMs) in healthcare communication. By evaluating this, we can determine how technological innovations can empower patients, addressing the challenges of understanding complex medical information in a patient-centered healthcare environment. This manuscript was posted as a preprint on the SSRN server [10].

### 2.1. Study Design

On ChatGPT 4.0 (February 2024 version), we employed prompts to translate three radiology report impressions into multiple target languages. The target languages were selected based on their prevalence in U.S. healthcare settings, reflecting the linguistic diversity encountered in hospitals and clinics. According to the U.S. Census Bureau, the top 5 most spoken non-English languages in the United States are Vietnamese, Tagalog, Spanish, Mandarin, and Arabic [11]. In addition, Hindi was used as a preliminary validation language due to the fluency of the investigators in this language, which allowed for an internal review of the survey tool. Four physicians proficient in ‘Hindi’ evaluated the translations for accuracy and clarity. The number of reports and physician translators used in the study was decided upon to perform the study in a limited period of time without any external funding.

We focused on three aspects of the responses generated by GPT-4:

1. Translation accuracy: the closeness of the translated text to the original English report.
2. Clarity of rephrased reports: how understandable was the rephrased radiology report for a patient or other non-medical person?
3. Patient comprehension: would a patient have a fair understanding of the simplified report?

### 2.2. Prompting

ChatGPT 4.0 was prompted with a specific command to directly translate each radiology report inputted. For the direct translation, the prompt used was, “Translate this radiology report about (the diagnoses) into (target language)”. After generating the direct translation, the same output was copied and pasted into the prompt box, and ChatGPT was asked to rephrase it using more straightforward layman’s terms. The prompt used was, “rephrase the translated report in layman’s terms so it is easier for a patient to understand”.

### 2.3. Selection of Radiology Reports

Three radiology reports of varying complexity were chosen to represent a spectrum of medical scenarios and complexity levels, providing a comprehensive assessment of ChatGPT 4.0’s capabilities. The three radiology report impressions used in this study were synthetically created by a practicing, American Board of Radiology-certified radiologist to ensure they represent a realistic spectrum of medical scenarios and complexities typically encountered in clinical practice. The reports do not represent real patient reports. The reports included common radiological assessments encountered in clinical practice, such as a mammogram, a CT scan for diverticulitis, and an MRI for hepatic metastases. The impression section of the report was copied and pasted into the prompt box for each report. The exact reports are as seen in Table 1.

### 2.4. Translation and Simplification Process

The selected radiology reports were input into ChatGPT 4.0, which performed the translation into the 5 chosen target languages and the subsequent simplification. The responses from ChatGPT 4.0 were captured verbatim for analysis.

### 2.5. Survey Design

A survey comprising of nine questions, three questions per report, was designed to evaluate the translations and simplifications. This survey was disseminated to MD physicians (via Google forms) who were native speakers of the target languages and could read the written text in the target language. The survey was sent to several physicians fluent in each language, aiming for at least three responses per language. The physician interpreters were also asked to record their specialty in one of the free text boxes. Each physician was asked to grade the ChatGPT 4.0 responses on a Likert Scale from 1 (worst) to 5 (best). The third question specifically addressed the understandability of the translation for a layperson. The questions on the survey are listed below:

- Question 1: Please rate the direct translation of the report from English to the target language on a scale of 1–5 (1—very inaccurate, 2—somewhat inaccurate, 3—moderately accurate, 4—very accurate, and 5—extremely accurate).
- Question 2: Please rate the simplified version of the report on a scale of 1–5 (1—very unclear, 2—somewhat unclear, 3—moderately clear, 4—very clear, and 5—extremely clear)
- Question 3: Would the patient have a fair understanding of their radiology report based on the rephrased translation (1—strongly disagree, 2—disagree, 3—neither agree or disagree, 4—agree, and 5—strongly agree)?

### 2.6. Data Analysis

For the analysis of the survey responses, we utilized descriptive statistics to summarize the data collected from the bilingual physician respondents. Each language group survey response was evaluated in terms of translation accuracy (question 1), clarity of the simplified report (question 2), and patient comprehension (question 3). The responses were visualized using bar charts to represent the distribution of scores across different languages and radiology reports. The bar charts were designed to display the percentage of respondents who strongly agreed/agreed, remained neutral, or disagreed/strongly disagreed for each question per report.

## 3. Results

A total of 24 native language expert physicians took part in the study and filled the online survey. The specialties of the physicians varied: three nephrologists, four internal medicine, four radiologists, one anesthesiologist, one immunologist, one psychiatrist, one sleep medicine, three cardiology, two critical/intensive care, two general medicine, and one family medicine. Four respondents were assessed in Hindi, five in Tagalog, six in Spanish, and three each in Vietnamese, Mandarin, and Arabic. Each response was recorded on a Likert scale of 1–5 (1—strongly disagree, 2—disagree, 3—neither agree or disagree, 4—agree, and 5—strongly agree). Table 2 provides a comprehensive presentation of all the physician responses collected during the study. The subsequent sections will offer a detailed analysis of the results for each language group.

### 3.1. Hindi

Hindi had 4 respondents. For report 1 (mammogram), 100% of participants (*n* = 4) agreed/strongly agreed with questions 1, 2, and 3, which asked about the direct translation, the translated simplified report, and understandability of said report.

Report 2 (diverticulitis) 75% of respondents (*n* = 3) agreed/strongly agreed about questions 1 and 2. A total of 100% (*n* = 4) agreed/strongly agreed with question 3.

**Figure 1:**
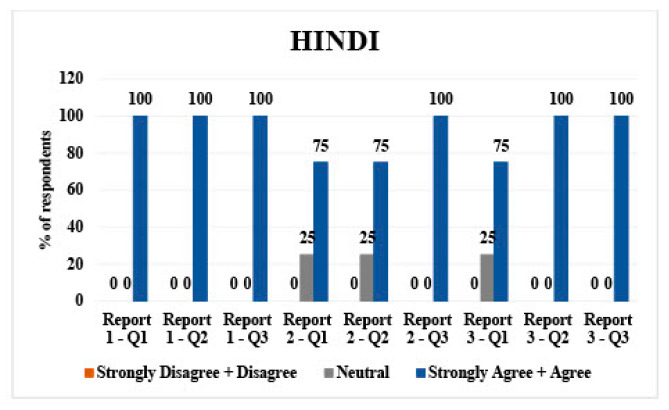
LLM Hindi validation bar chart.

For report 3 (hepatic metastases), 75% of respondents (*n* = 3) agreed/strongly agreed for question 1. A total of 100% (*n* = 4) agreed with questions 2 and 3 (Figure 1).

### 3.2. Tagalog

Tagalog had five respondents. For report 1, 60% (*n* = 3) respondents were neutral to questions 1 (direct translation) and 2 (simplified translation), and 80% (*n* = 4) strongly agreed/agreed with question 3 (understandability of translations).

For report 2, 60% (*n* = 3) respondents were neutral to question 1, 80% (*n* = 4) were neutral to question 2, and 60% (*n* = 3) respondents strongly agreed/agreed with question 3.

**Figure 2:**
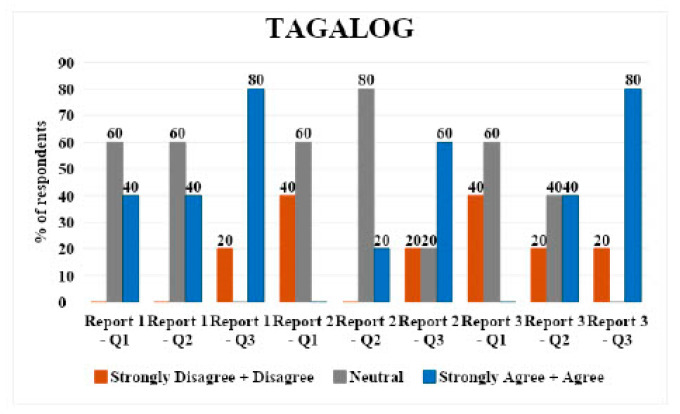
LLM Tagalog language validation bar chart.

For report 3, 60% (*n* = 3) respondents were neutral to question 1, 40% (*n* = 2) strongly agreed/agreed with question 2, and 80% (*n* = 4) strongly agreed/agreed with question 3. Overall, there were mixed responses with a tendency towards neutrality in direct translations and simplified versions across all reports (Figure 2).

### 3.3. Spanish

Spanish had six respondents; 16.7% (*n* = 1) disagreed/strongly disagreed with questions 1, 2, and 3 of report 1. Neutral opinion was given by 16.7% (*n* = 1 respondent) for the direct translation of report 1. The number of respondents who agreed/strongly agreed with the direct translation, simplified version of the report, as well as understandability of report 1 were 66.7% (*n* = 4), 66.7% (*n* = 5), 83.3% (*n* = 5), and 83.3% (*n* = 5), respectively.

In report 2, 16.7% (*n* = 1) were neutral and 83.3% (*n* = 5) agreed/strongly agreed with the direct translation; 33.3% (*n* = 2) disagreed/strongly disagreed and 66.7% (*n* = 4) agreed/strongly agreed with the simplified version of translation; and 33.3% (*n* = 2) disagreed/strongly disagreed, 16.7% (*n* = 1) was neutral, and 50% (*n* = 3) agreed/strongly agreed with understandability of the simplified report.

**Figure 3:**
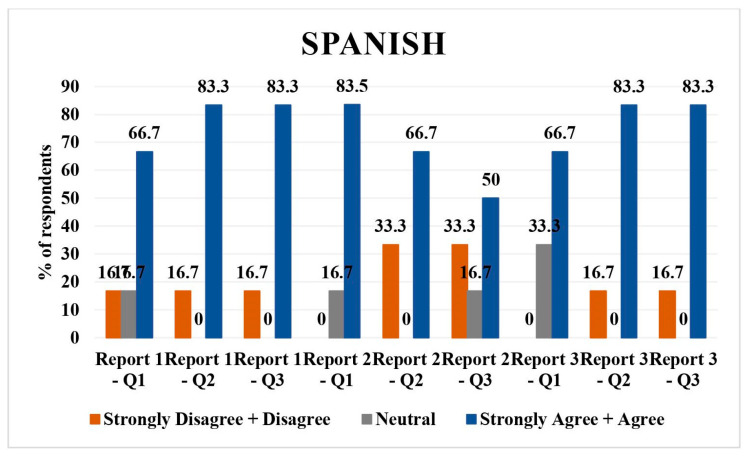
LLM Spanish language validation bar chart.

Regarding report 3, 33.3% (*n* = 2) had neutral opinion and 66.7% (*n* = 4) agreed with the direct translation. Concerning simplified translation of the report and understandability of the simplified report, 16.7% (*n* = 1) disagreed and 83.3% (*n* = 5) agreed. Overall, the majority agreed/strongly agreed with translations and understandability, with some disagreement in report 2 (Figure 3).

### 3.4. Vietnamese

The Vietnamese language had three respondents. In report 1 translation, 33.3% (*n* = 1) disagreed/strongly disagreed and 66.7% (*n* = 2) were neutral regarding the direct translation of report. As for the simplified translation of report 1, 66.7% (*n* = 2) were neutral and 33.3% (*n* = 1) agreed/strongly agreed with it; 66.7% (*n* = 2) disagreed/strongly disagreed and 33.3% (*n* = 1) agreed/strongly agreed to the understandability of the report.

A total of 66.7% (*n* = 2) and 33.3% (*n* = 1) disagreed/strongly disagreed with the direct translation and understandability of simplified report 2, and 66.7% (*n* = 2) were neutral to the simplified translation of report 2.

**Figure 4:**
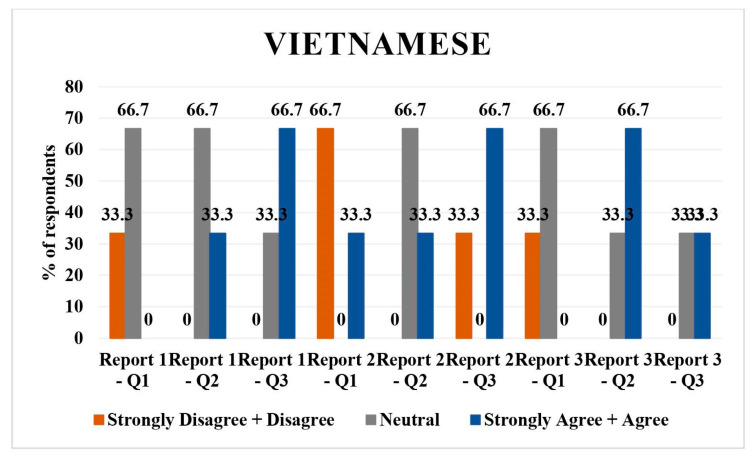
LLM Vietnamese language validation bar chart.

A total of 33.3% (*n* = 1), 33.3% (*n* = 1), and 66.7% (*n* = 2) agreed/strongly agreed to the direct translation, simplified translation, and understandability of the simplified report 3. Predominantly neutral or disagreeing responses were seen on direct translations and simplified versions (Figure 4).

### 3.5. Mandarin

There were three respondents for Mandarin. For report 1, 100% (*n* = 3) agreed/strongly agreed with the direct translation and simplified translation. Regarding the understanding of the simplified report, 33.3% (*n* = 1) were neutral and 66.7% (*n* = 2) agreed/strongly agreed with it.

In the report 2 assessment, 33.3% (*n* = 1) were neutral and 66.7% (*n* = 2) agreed/strongly agreed with direct translation and simplified translation. All the respondents agreed/strongly agreed with understandability of simplified report 2 (*n* = 3).

**Figure 5:**
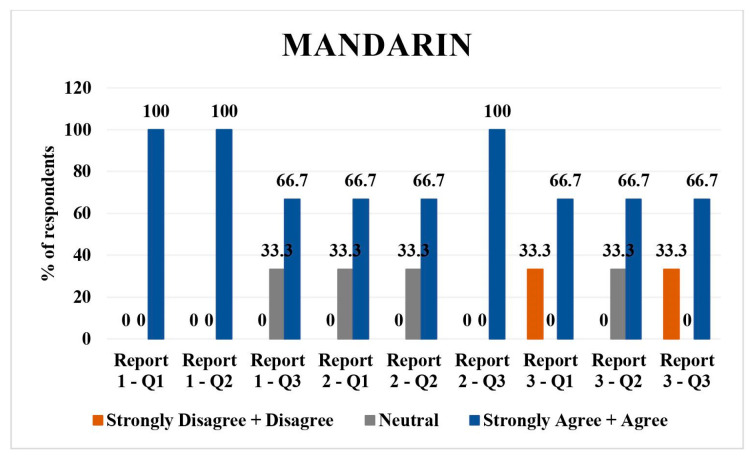
LLM Mandarin language validation bar chart.

A total of 33.3% (*n* = 1) strongly disagreed/disagreed with the direct translation of report 3, whereas 66.7% (*n* = 2) agreed/strongly agreed with it. Similar responses were recorded for question 3 of report 3. For the simplified version of report 3, 33.3% (*n* = 1) were neutral and 66.7% (*n* = 2) agreed/strongly agreed to the report. Overall, there was high agreement/strong agreement on direct translations and understandability (Figure 5).

### 3.6. Arabic

Arabic had three respondents. Regarding report 1, 33.3% (*n* = 1) each disagreed/strongly disagreed, were neutral, and agreed/strongly agreed with the direct translation. In total, 33.3% (*n* = 1) were neutral and 66.7 (*n* = 2) agreed/strongly agreed with the simplified translation of report 1. In total, 100% (*n* = 3) agreed/strongly agreed with the understandability of the report.

Report 2 had 66.7% (*n* = 2) disagreed/strongly disagreed, while 33.3% (*n* = 1) agreed/strongly agreed with direct translation. In total, 100% (*n* = 3) agreed/strongly agreed with the simplified translation and understandability of the simplified report.

**Figure 6:**
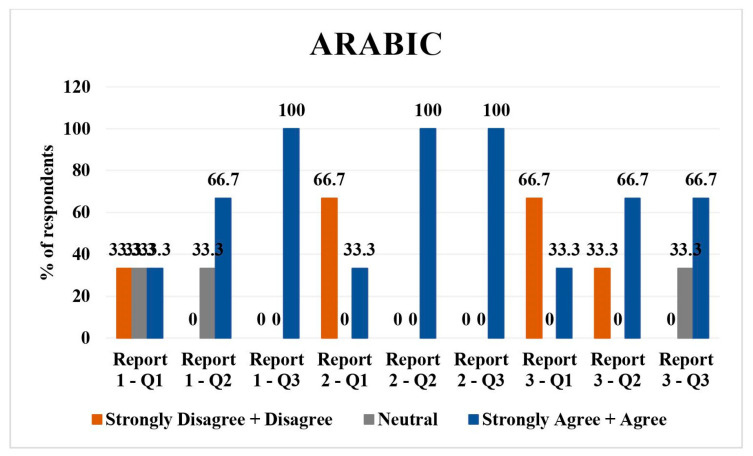
LLM Arabic language validation bar chart.

For report 3, 66.7% (*n* = 2) disagreed/strongly disagreed and 33.3% (*n* = 1) agreed/strongly agreed with direct translation of the report. In total, 33.3% (*n* = 1) disagreed/strongly disagreed 66.7% (*n* = 2) agreed/strongly agreed with the simplified version of the report. Regarding understandability of the simplified report, 33.3% (*n* = 1) were neutral and 66.7% (*n* = 2) agreed/strongly agreed. Overall, there were mixed responses, with more disagreement on direct translations but higher agreement on simplified versions and their understandability (Figure 6).

### 3.7. Overall Findings

- Hindi: all respondents rated the direct translations, simplified versions, and their understandability as agreeable/strongly agreeable, except for one neutral opinion on the direct translation of report 2 and 3.
- Tagalog: Mixed responses with a tendency towards neutrality in direct translations and simplified versions across all reports. A slight disagreement was noted in simplified versions, particularly for report 3.
- Spanish: majority agreed/strongly agreed with translations and understandability, with some disagreement or neutrality, particularly on the simplified versions of reports 1 and 2.
- Vietnamese: predominantly neutral or disagreeing responses on direct translations and simplified versions, with a more positive response for report 3.
- Mandarin: high agreement/strong agreement on direct translations and understandability, with one neutral and one disagreeing response on the direct translation of report 3.
- Arabic: mixed responses, with more disagreement on direct translations but higher agreement on simplified versions and their understandability.

## 4. Discussion

The AI-MIRACLE study reveals insights into the application of large language models (LLMs), specifically ChatGPT 4.0, in healthcare communication, particularly in translating and simplifying English radiology reports into multiple languages. Specifically, reports related to breast mammograms (report 1), diverticulitis (report 2), and hepatic metastases (report 3) each posed different challenges and levels of success in translation, as evidenced by the variable responses from our physician respondents. Our findings reveal the heterogeneous performance of ChatGPT 4.0 across different languages, underscoring both the potential and the limitations of current AI technology in the realm of translating medical reports, as was noted in a recent study by Gulati et al. [12].

In line with previous research indicating the utility of AI in enhancing patient comprehension of medical documents [2], our study found that the Hindi translations and simplifications were particularly effective, as evidenced by the high level of agreement among native Hindi-speaking physicians. A similar study assessing the translation of radiology reports into simpler words showed promising results, with a low frequency of missing or incorrect information and high overall scores [13]. Interestingly, a study by Sarangi et al., showed that direct translations of radiology reports into Hindi were fairly accurate, but summarized reports in layman terms were not suitable for patient communication [14]. This discrepancy may have been due to the different versions of ChatGPT being used. Their study used the free version (ChatGPT 3.5), whereas we used the paid and updated version, ChatGPT4.

The Spanish translations were largely successful, with some disagreements in the direct translation of reports suggesting that ChatGPT translations can be useful for relaying the complex radiology reports in simpler words for patients who prefer Spanish [9]. There were no prior direct studies discussing radiology report translation to Spanish. However, several studies corroborate that ChatGPT can successfully translate English reports into simpler layman terms for easier understanding [15].

There was variability in results with the Tagalog and Vietnamese languages, with more agreement on the simplified version of the report than the direct translations in Tagalog. Vietnamese had mixed responses with predominantly neutral responses. These mixed responses may have been due to a lack of direct training data in these languages due to the dearth of medical and radiological literature in these languages, indicating that even LLMs such as GPT-4 need to be better suited for low-resource languages. A study by Fang et al. [16] assessed ChatGPT performance in Mandarin medical exams and found that it was satisfactory but needed improvement when posed with non-English medical text. Some of the commercially available medical translation services for English to Vietnamese and Tagalog stress having highly trained and certified translators due to the significant complexity of these languages [17].

Mandarin translations were well received with the majority agreed with the direct translation and simplification of the radiology reports. These findings were similar to the satisfactory performance of ChatGPT on Mandarin medical licensing examination. Another study by Liu et al., showed satisfactory performance of AI and LLMs in creating a parallel corpus for English–Chinese translation in the biomedical domain [18].

Arabic translations demonstrated mixed responses, with more disagreement on direct translations but higher agreement on simplified versions and their understandability. A study by Sahari et al., studied the use of ChatGPT in translation in an Arab context and they also found that ChatGPT performed well for more straightforward translations compared to complex medical text [19]. Another study by Khoshafa et al., showed that ChatGPT can be used as a translator for simple content. However, human intervention is still required, especially for complex texts that ChatGPT is unable to correctly interpret and translate [20].

The strengths of our study include being the first to employ LLMs for translating radiology reports into the top five non-English spoken languages in the United States [11]. The respondents were practicing physicians who could read the target language, enhancing the accuracy of their responses. The radiology reports were created by one of the authors who is an attending radiologist, demonstrating a spectrum of some of the most common radiology exam types, such as screening mammogram, CT abdomen for acute diverticulitis, and MRI abdomen demonstrating cancer follow-up exam. The senior authors of this manuscript have past experience of evaluating large language model in the field of radiology [2] and critical care medicine [21].

Some of the limitations include the difference in the number of physician respondents for each language assessed, which varies from three to six. This variation was unintended and a consequence of higher-than-expected response rates (originally planned for three per language), which could contribute to potential biases or limitations in our analysis. One of the other limitations of our study is that the small number of radiology reports may not adequately represent the complexity and variety of radiology reports encountered in clinical practice. Future studies should include a larger and more diverse set of reports to enhance the generalizability of the findings. Although a necessary ethical consideration, synthetic radiology reports might not reflect the full complexity of genuine patient reports. Despite this, the findings offer an important initial insight into the strengths and weaknesses of current language learning models in the context of medical translational tasks. Another limitation is that ChatGPT is not explicitly designed for medical report translation and is more of a generic LLM. Other general limitations of LLMs are that they have a tendency to agree if pressed hard, hallucinations, inherent algorithm biases, and investigator confirmation bias may apply while replicating similar studies. Future studies can address whether some parts of the reports were translated better than others and other such patterns can be identified.

## 5. Conclusions

The AI-MIRACLE study offers novel insights into the application of large language models, such as ChatGPT 4.0, in the domain of healthcare communication. Our study demonstrates that ChatGPT 4.0 can play a significant role in breaking language barriers in healthcare, potentially improving patient comprehension of complex medical information. However, there are some challenges associated with such technological applications. The performance of ChatGPT 4.0 in translating and simplifying medical texts was found to be heterogeneous across different languages, indicating a gap in the model’s training or limitations in handling certain low resource languages. This variability highlights the need for continued refinement in AI technology, with more inclusive and extensive training datasets. Moving forward, there is a clear pathway for the advancement of AI in healthcare communication. Future research should concentrate on enhancing the capabilities of AI systems in dealing with a diverse range of languages and medical contexts. Addressing our studies’ limitations will enhance the understanding and utilization of AI in healthcare communication, ultimately improving patient comprehension and outcomes.

## Figures and Tables

**Table 1:** Details of the 3 Radiology Reports Used for LLM Validation.

Report 1 (Mammogram)	Report 2 (Diverticulitis)	Report 3 (Hepatic Metastases)
Left breast focal asymmetry with questionable distortion. No evidence of malignancy in the right breast. BI-RADS Category 0: Incomplete: Need additional imaging evaluation and/or prior mammograms for comparison. RECOMMENDATIONS: Recommend further evaluation of the left breast with 2D/3D 90 degree lateral imaging and CC/MLO spot compression imaging with additional mammographic views and ultrasound if needed. A second reading of the examination was performed using computer-aided detection (CAD).	Diffuse mucosal thickening with enhancement involving the sigmoid colon, with a background of diverticulitis. Focal area of fat stranding and small amount of fluid is seen adjacent to the sigmoid colonic diverticulum represents acute sigmoid diverticulitis, with small adjacent fluid collection measuring up to 1.7 × 1.8 cm which represents a small abscess. Recommend follow up CT abdomen and pelvis with and without contrast in 3 months. Correlate clinically.	Interval progression of the metastatic lesion in segment V of liver, which now measures approximately 3.5 × 3.2 cm, increased from the prior 1.7 × 1.5 cm. Additionally, there is interval appearance of a new 1.8 cm peripherally enhancing lesion in segment VIII of liver compatible with a new metastatic deposit. Overall, findings represent worsening hepatic metastatic disease burden.

**Table 2:** Comprehensive Summary of Physician Responses Across Language Groups and Questions (% Agreement on Likert Scale).

Report	Language	Question	1. Strongly Disagree	2. Disagree	3. Neutral	4. Agree	5. Strongly Agree
Report 1	Arabic	Q1		33.3	33.3		33.3
		Q2			33.3	33.3	33.3
		Q3				100.0	
	Mandarin	Q1				33.3	66.7
		Q2				66.7	33.3
		Q3			33.3		66.7
	Spanish	Q1	16.7		16.7	16.7	50.0
		Q2		16.7		66.7	16.7
		Q3		16.7		33.3	50.0
	Tagalog	Q1			60.0	40.0	
		Q2			60.0	40.0	
		Q3		20.0		80.0	
	Vietnamese	Q1		33.3	66.7		
		Q2			66.7	33.3	
		Q3			33.3	66.7	
Report 2	Arabic	Q1		66.7		33.3	
		Q2				66.7	33.3
		Q3				33.3	66.7
	Mandarin	Q1			33.3	33.3	33.3
		Q2			33.3	33.3	33.3
		Q3				66.7	33.3
	Spanish	Q1			16.7	33.3	50.0
		Q2		33.3		50.0	16.7
		Q3		33.3	16.7	16.7	33.3
	Tagalog	Q1	20.0	20.0	60.0		
		Q2			80.0	20.0	
		Q3		20.0	20.0	60.0	
	Vietnamese	Q1		66.7		33.3	
		Q2			66.7	33.3	
		Q3		33.3		66.7	
Report 3	Arabic	Q1	33.3	33.3			33.3
		Q2		33.3		33.3	33.3
		Q3			33.3	33.3	33.3
	Mandarin	Q1		33.3		33.3	33.3
		Q2			33.3	33.3	33.3
		Q3		33.3		33.3	33.3
	Spanish	Q1			33.3	16.7	50.0
		Q2		16.7		33.3	50.0
		Q3		16.7		33.3	50.0
	Tagalog	Q1	20.0	20.0	60.0		
		Q2		20.0	40.0	40.0	
		Q3		20.0		60.0	20.0
	Vietnamese	Q1		33.3	66.7		
		Q2			33.3	66.7	
		Q3			33.3	33.3	33.3

## Data Availability

All data analyzed during this study are included in the published article.
